# Prediction of cardiac cycle duration for cardiac-gated closed-loop auricular vagus nerve stimulation

**DOI:** 10.3389/fnins.2026.1869668

**Published:** 2026-06-26

**Authors:** Dibya Chowdhury, Kevin Schuh, Eugenijus Kaniusas

**Affiliations:** Institute of Biomedical Electronics, Vienna University of Technology, Vienna, Austria

**Keywords:** auricular vagus nerve stimulation, cardiac-gated stimulation, ECG, heartbeat prediction methods, personalized neuromodulation

## Abstract

Auricular vagus nerve stimulation (aVNS) is a neuromodulation technology that establishes balance in the autonomic nervous system and, in turn, provides therapy for numerous chronic ailments. Personalized aVNS adapts the stimulation parameters in accordance with the time-varying physiological state of the body, and is suggested to improve the therapeutic outcomes and reduce side effects. The physiological state is estimated via recorded biomarkers such as the electrocardiogram (ECG). aVNS can be delivered in synchrony with any phase of the cardiac cycle before and after the R-peak. This paper proposes the prediction of the duration of the next cardiac cycle after the detected R-peak for the realization of the personalized cardiac-gated closed-loop aVNS applied at any time point during the predicted cardiac cycle. We propose and explore the feasibility of four different prediction methods for predicting the duration of the next cardiac cycle. Two methods are respiration-insensitive, last value and averaging, and the other two are respiration-sensitive, extrapolation and interpolation. Offline recorded ECG waveforms were used to evaluate the different methods. Subsequently, three of the four methods (last value, averaging, and extrapolation) were implemented in real-time on a proprietary aVNS hardware setup, with the data acquisition performed across normal and paced deep breathing. Offline evaluation of the methods revealed that extrapolation and interpolation achieved lower prediction errors during deep breathing with the median absolute error (MdAE) of 32.09 ms (interquartile range 16.07–56.61 ms) and 31.71 ms (15.5–54.06 ms), respectively, as compared with the averaging and last-value methods with 88.75 ms (58.73–124.15 ms) and 40.85 ms (19.7–68.4 ms), respectively. During normal breathing, all evaluated methods yielded lower prediction errors relative to the averaging method 28.5 ms (15.2–43.7 ms). Real-time implementation validated these methods for closed-loop cardiac-gated aVNS, with the best performance achieved by the extrapolation method with 31.4 ms (15.17–55.9 ms) during paced deep breathing. During normal breathing, comparable performance across prediction methods favors the computationally simple last-value approach (MdAE: 31.6 ms). Proposed methods establish the potential of ECG-based R-peak prediction in real-time as a reliable and individual biomarker for the personalized cardiac-gated aVNS, creating a foundation for future clinical applications of aVNS.

## 1 Introduction

Vagus nerve stimulation (VNS) is the neuromodulation technique that exhibits therapeutic effects for a spectrum of pathological conditions, owing to the vagus nerve's direct innervation of thoracic and abdominal organs, its involvement in the autonomic control, sympathovagal balance, brain plasticity, immune function and others (Groves and Brown, 2005), (Kaniusas et al., 2019b). The vagus nerve governs parasympathetic activity, facilitates interaction between the central nervous system and, on the other hand, the cardiovascular, the respiratory, and gastrointestinal systems, supporting VNS therapeutic potential in these systems (Kaniusas et al., 2019b).

### 1.1 From invasive to non-invasive VNS

The administration of VNS has been implemented using both invasive and non-invasive methods. The invasive methods, involving implanted electrodes, have been established for therapeutic intervention in epilepsy and depression (Ravan, 2017; Edwards et al., 2017). The non-invasive method of VNS have gained prominence due to their low cost and minimal risk, providing therapy for medical conditions in fields such as cardiology, neurology, psychology, and metabolic wellness, while also mitigating pain (Huang et al., 2016; Garcia et al., 2017; Keute et al., 2021).

An external branch of the afferent vagus nerve, with a direct projection to the brainstem, is present in the outer ear, making it readily accessible for the non-invasive stimulation (Mercante et al., 2018). Surface or miniature needle electrodes are positioned in the vagally innervated areas of the ear (such as cymba concha) and subjected to electrical stimulation. The auricular VNS (aVNS) has garnered increasing scientific interest due to its easily accessible location, thus holding considerable potential in reestablishing sympathovagal balance by improving brain plasticity (Kampusch et al., 2015). Over the years, several clinical applications of aVNS have been reported, including reductions in the magnitude and duration of epileptic seizures (Bauer et al., 2016), symptomatic relief in depression (Rong et al., 2016), and improved functional recovery in stroke rehabilitation (Capone et al., 2017). The therapeutic potential of aVNS is shown in cardiovascular diseases (Ottaviani et al., 2022), such as the reduction of blood pressure in pre-diabetic patients (Huang et al., 2016).

### 1.2 From open-loop to closed-loop VNS

Therapeutic stimulation parameters, including intensity and on/off cycles, are typically individualized according to the patient's perception and tolerance to stimulation, with stimulation patterns usually determined by the attending physician at the initiation of therapy (Greif et al., 2002). Continuous aVNS, empirically determined, was provided over a certain duration as a therapeutic intervention (Straube et al., 2015). Regardless of the reported benefits of aVNS, being an open-loop pathway, there is still an ambiguity due to a lack of individualized control over the stimulation parameters. This may lead to overstimulation or become a non-responder, not catering to the therapy appropriately (Ottaviani et al., 2022).

Absence of temporal variation in intensity and incidence according to the current physiological state of the individual undergoing aVNS therapy prompted the need for a closed-loop aVNS. Adaptive control of aVNS requires biofeedback, which reflects the individual's instantaneous physiological state, allowing stimulation parameters to adjust dynamically according to the person's current condition (Mylavarapu et al., 2023) and pathological state (Kaniusas et al., 2019a). As a result, efforts and studies have been conducted over the years to determine the responsiveness of various biomarkers to aVNS (Keute et al., 2021; Rapalis et al., 2023). The earliest closed-loop vagus nerve stimulation was used to treat epileptic seizures based on heart rate monitoring (Sun and Morrell, 2014). Here VNS was synchronized with the movement during rehabilitation exercises in stroke patients using electromyogram, resulting in improved rehabilitation outcomes (Epperson et al., 2024). Another study on the closed-loop aVNS utilized electromyogram biomarkers by detecting activity from the orofacial muscles of neonates, while simultaneously delivering stimulation to facilitate motor rehabilitation (Cook et al., 2020). The electroencephalogram served as a biomarker for closed-loop aVNS targeting the delta phase to enhance therapeutic outcomes for delirium, with the prototype tested in healthy volunteers (Anzolin et al., 2023). Respiration has also been employed as a biofeedback signal to guide closed-loop aVNS, since vagal activity is gated by the respiration cycle. They triggered the stimulation during the expiration phase, with concurrent real-time monitoring of heart rate (de Faria et al., 2024). In fact, respiration (especially, deep breathing) and aVNS individually enhance their neuromodulatory effects on the brainstem, because respiration-modulated signals (e.g., from pulmonary stretch receptors and baroreceptors) converge with the afferent signals from aVNS in the brainstem, leading to stronger modulation of parasympathetic networks than either intervention could achieve alone (Garcia et al., 2017). Nevertheless, implementing a truly personalized closed-loop aVNS system poses significant challenges, particularly due to variations in biomarkers that depend on the patient's condition.

### 1.3 Biomarker and their prediction

Biomarkers for VNS are generally derived from autonomic biomarkers such as cardiac cycle and heart rate variability, respiration cycle, saliva composition, pupil diameter and reflex, and electroencephalography (Warren et al., 2019; Konakoğlu et al., 2023; Bömmer et al., 2024). The electrocardiogram (ECG) emerges as a promising marker, representing the electrical function of the heart. It is inexpensive, safe to record, and shows marked responsiveness to aVNS (Gurel et al., 2020) for monitoring cardiovascular diseases (Ottaviani et al., 2022). ECG-synchronized aVNS has been suggested in a prior study as a strategy for temporally controlled stimulation in future studies (Yu et al., 2022) and experimentally validated in our recent study (Tischer et al., 2024). Personalized, minimally invasive aVNS approaches utilizing ECG and photoplethysmography as biomarkers have also been developed by our group, demonstrating the feasibility of cardiac-gated aVNS controlled by electrical and mechanical activity of the heart, respectively (Dabiri et al., 2022), with the cardiac activity also gated by the respiratory cycle. Furthermore, studies have demonstrated that the precise timing of aVNS within the cardiac cycle, e.g., timing with respect to the R peak of ECG, can impact autonomic efferent cardiovagal responses, namely the aVNS-guided heart's acceleration and deceleration, thereby supporting the suitability of ECG as a potentially reliable biomarker (Tischer et al., 2024). In particular, systole-gated aVNS coincides with the natural burst of vagal afferent firing driven by arterial baroreceptors, whereas diastole represents an inactive or in relaxation baroreceptor window (Karemaker, 2022; Tischer et al., 2024).

Alongside the use of ECG-based biofeedback for closed-loop aVNS, identifying the precise point of stimulation within the cardiac cycle and a timely application of aVNS became essential. However, due to intrinsic and unavoidable delays in real-time ECG recording, processing, and formation of aVNS stimulus, an advanced prediction of the cardiac cycle duration becomes necessary (Zacarias et al., 2024). All the more, stimulation before the fiducial point in ECG is essentially not possible. In the study of ECG-based aVNS (Tischer et al., 2024), the authors evaluated heart rate responses to percutaneous aVNS delivered flexibly during either the systolic or diastolic phase of the ECG cycle, before and after the fiducial point, highlighting the need for predicting the duration of the next cardiac cycle. For cardiac cycle prediction, sophisticated time-series forecasting models, notably the AutoRegressive Integrated Moving Average (ARIMA) and Kalman-filter approaches—have been widely utilized for RR interval tracking. For instance, optimized hybrid ARIMA-GARCH architectures have been successfully applied to RR interval volatility and preserve data trends for disease monitoring (Shu, 2024) with a mean absolute with a mean absolute percentage error of maximum 4.36%. Likewise Kalman filters have been used as Bayesian estimation procedure to estimate prediction errors in RR interval ranging from -100 ms to 100 ms. (Hirao et al., 2026). While there have been some advances in the prediction of future cardiac cycles *in silico*, real-time prediction of the subsequent cardiac cycle poses a certain level of complexity (Zacarias et al., 2024), (Branen et al., 2022).

We propose, evaluate and analyze four different prediction methods to predict the next RR interval from the ECG offline and in real-time in order to apply aVNS pulses at an arbitrary time point within the cardiac cycle (Kaniusas et al., 2020). In particular, the feasibility of the predictive targeting of the next R peak is evaluated. An overview of the prediction methods is presented in Section II, their analysis is reported in Sections III and IV and the study is concluded in Section V.

## 2 Materials and methods

### 2.1 Research design and approach

**Figure 1:**
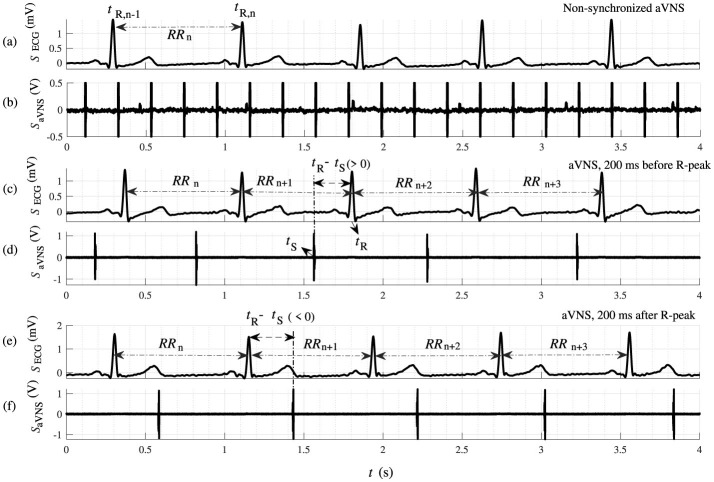
Non-gated and cardiac-gated aVNS **(a)** ECG signal, which is not synchronized with **(b)** aVNS stimulus applied at every 200 ms. **(c, d)** The cardiac-gated aVNS applied with the time delay *t*_R_−*t*_S_ = 200 ms (>0) before R-peak during diastole, and **(e, f)** with *t*_R_−*t*_S_ = −200 ms (< 0) after the R-peak during systole using the introduced extrapolation method.

The present pilot study implements real-time prediction of ECG R-peaks and utilizes it for the personalized, cardiac-gated aVNS; however, without aVNS electrodes connected to the ear (Figure 1). ECG data for the offline and real-time settings were taken from and recorded within the pre-clinical pilot study, which was approved by the institutional review board of the Medical University Vienna (EK NR 2121/2022, entitled: “Cardiac-gated auricular vagus nerve stimulation in a closed-loop biofeedback system, for adaptive stimulation in healthy subjects—pilot study” and published in (Tischer et al. 2024) with the informed consent being signed by all participants.

For normal breathing and offline settings, 5-min (in total 1,252 RR intervals) data was recorded from three healthy subjects (age 30, 21, 22, with 2 females) in a quite seated position. For paced deep breathing and real-time settings, serving as reference with respect to strengthened respiratory modulation of the heartbeat interval and hardware restrictions, respectively, 5-min (in total 345 RR intervals) data was taken from only one subject (aged 30 years, female).

To ensure the robustness and validation of the prediction methods based on independent data, due to our limited in-house cohort, ECG data were taken from 10 healthy subjects (4 males and 6 females; aged 22–30 years, 5-min each, in total 3357 RR intervals) sourced from the Combined measurement of ECG, Breathing and Seismocardiograms Database (CEBSDB) via the open-source PhysioNet repository (García-González et al., 2013; Goldberger et al., 2000). Furthermore, to evaluate the boundaries of prediction methods during an abnormal autonomic state, RR intervals were taken from 2 subjects with tachycardia (both males, aged 30 and 31, 5-min each, in total 784 RR intervals), sourced from Spontaneous Ventricular Tachyarrhythmia Database via PhysioNet (Goldberger et al., 2000).

#### 2.1.1 Offline setting

In the offline setting, ECG data (lead 1 of Einthoven triangle, sampling frequency of 1 kHz) recorded for 5 min is processed with MATLAB under two conditions: normal, spontaneous breathing and consciously paced deep breathing, where the subject takes deep, continuous breaths at a comfortable, consciously slowed pace with the resulting period of about 6 seconds. Cardiac cycles are determined by detecting the R-peaks in the recorded ECG signal and are represented by the resulting RR values. A combined slope and amplitude-based detection approach is used to identify the temporal location of the R-peaks. For the amplitude-based approach, a threshold is set, and the ECG samples are compared against this set threshold. ECG samples with amplitudes greater than the threshold are identified as potential locations for R-peaks. Subsequently, for the slope-based step, the slope in ECG is calculated and compared with the slope threshold. The QRS complex of the ECG, having a peak amplitude and a steeper slope compared to the P and T waves, thus helps to co-determine the true R-peak, which is then used for further analysis of the R-peak prediction methods (Section 2.2). All detected peaks were visually controlled, and the absence of extrasystoles was assured.

Respiratory phases, i.e., inspiration and expiration, were derived directly from the ECG signal by tracking the continuous shortening and widening, respectively, of the RR intervals driven by Respiratory Sinus Arrhythmia (RSA), without using any additional respiratory sensor to minimize hardware complexity and computational overhead.

#### 2.1.2 Real-time setting

The closed-loop cardiac-gated aVNS setup for the real-time setup is composed of two functional modules: (1) a biosignal acquisition system (BIOPAC-MP36 system), where ECG (lead 1 of the Einthoven triangle) can be acquired from the individual and simultaneously transmitted to other systems (here to the aVNS stimulator). (2) A proprietary aVNS stimulator (based on STM32G474RE, ArmCortex-M4 MCU- with clock frequency 170MHz, with 512 Kbytes of flash memory, 4 KB of SRAM, largely dedicated to maintaining the adaptive circular buffers) is used. The hardware's ADC acquires the analog incoming ECG signal in real time, and notes the RR interval when an interrupt is triggered. The RR intervals are saved in circular buffers. Following this the prediction methods are executed and output voltage stimulus is triggered simultaneously in real-time to a resistive load of 5 kΩ (mimicking the resistance of the human ear).

Considering aVNS, a triphasic stimulation pattern was generated with a duration of 6 ms, a pulse width of 1 ms, and a magnitude of 1 V or 0.5 V (Kaniusas et al., 2020). The aVNS output was then fed back into the BIOPAC system for analysis. Thus, ECG and aVNS, delivered according to the individual's ECG in a time-gated manner, are both recorded in real-time and synchrony by the BIOPAC system.

The processed ECG data are utilized in aVNS stimulator to derive personalized stimulation parameters for the adaptive aVNS control. For preliminary validation of the prediction methods in real-time, two 5-min closed-loop datasets were recorded. aVNS was delivered in synchrony and with a targeted phase shift relative to the predicted R-peaks of the cardiac cycle in real-time under both normal and paced deep breathing conditions.

For the evaluation of the real-time setting, the time points *t*_R_ and *t*_S_ are introduced (Figures 1c, d). The time *t*_R_ denotes the time instances of the true R-peaks of the ECG that were to be predicted. The time *t*_S_ denotes the time instance of the applied aVNS stimulus Δ*t* (set to 200 ms in this study) before or after *t*_R_, where Δ*t* can be assigned any value by the researcher or caregiver depending on, for instance, whether systole-gated or diastole-gated aVNS should be performed. Correspondingly the difference *t*_R_−*t*_S_ describes the time shift of the stimulus with respect to the predicted R-peak, whereas the prediction error (*t*_R_−*t*_S_−Δ*t* ms) describes how precisely the proposed methods provide aVNS stimulus Δ*t* ms before the predicted R-peak.

#### 2.1.3 Methods

The detected R-peaks are used to predict the next R-peak which serves as a reference for administering aVNS, before or after the predicted R-peak, ensuring synchronization with any arbitrary phase of the cardiac cycle and thus, establishing a closed-loop aVNS system.

Four prediction methods: (a) last value, (b) averaging, (c) extrapolation and (d) interpolation, are designed and analyzed for the prediction of the next ECG R-peak. Following the offline implementation of the prediction methods, they were embedded in the proprietary hardware platform in real-time. In this real-time setting, only three methods (excluding the interpolation method) were tested for prediction, given the hardware restrictions of the proprietary aVNS stimulator.

### 2.2 Prediction methods

For predicting cardiac cycles for closed-loop control of aVNS, a circular storage buffer, is used to store the real-time *RR*_R_ values of a single respiratory cycle. The obtained R-peaks samples are used to calculate the actual *RR*_R_ interval (Equation 1 and Figure 1a).


$$\begin{array}{l} RR_{\text{R},\text{n}}=t_{\text{R},\text{n}}-t_{\text{R},\text{n}-1} \end{array}$$ (1)

Here, *t*_R, n_ and *t*_R, n − 1_ are the current and preceding temporal location of R-peaks. These *RR*_R_ intervals are then used to predict the next RR interval *RR*_P_ using four proposed methods. Methods 1 and 2 predict the *RR*_P_ without accounting for the influence of respiration, whereas methods 3 and 4 consider the impact of cyclic respiration on the RR sequence and predict the next cardiac cycle accordingly.

#### 2.2.1 Last value

In the last value method, the predicted RR interval, $RR_{\text{P},\text{n}+1}^{1}$ at (*n*+1)^*th*^ time instant is mathematically identical to the duration of the most recently completed cardiac cycle *RR*_R_ (at the sampling instant *n*) (Equation 2).


$$\begin{array}{l} RR_{\text{P},\text{n}+1}^{1}=RR_{\text{R},\text{n}} \end{array}$$ (2)

#### 2.2.2 Averaging

The predicted RR interval $RR_{\text{P},\text{n}+1}^{2}$ is the average of preceding *N* *RR*_R_ intervals, computed by summing *RR*_*R*_(*n*) back to *RR*_*R*_(*n*−*N*) and dividing by the fixed window length *N*, providing a short-term smoothed estimate of the future *RR*_P_ interval. In this experiment, *N* was set to 4 to consider a reasonable fraction of the respiration cycle (Equation 3).


$$\begin{array}{l} RR_{\text{P},\text{n}+1}^{2}=\frac{\overset{\text{n}-\text{N}+1}{\underset{\text{i}=\text{n}}{\sum }}RR_{\text{R}\left(\text{i}\right)}}{N} \end{array}$$ (3)

#### 2.2.3 Extrapolation

**Figure 2:**
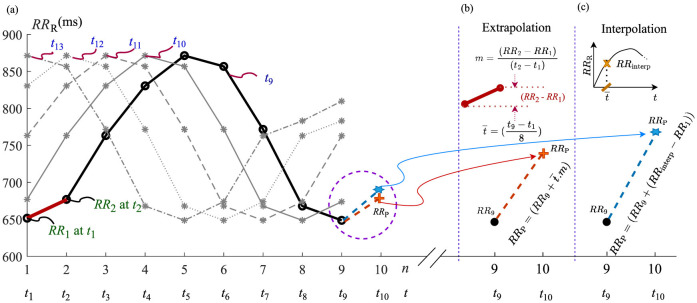
**(a)** Real inter-beat *RR*_R_ intervals within a single and full previous respiration cycle during paced deep breathing with a respiration period equaling *t*_9_−*t*_1_ at the current time point *t*_9_ (solid black line). The x- axis represents a discrete cardiac cycle index for the momentary respiratory cycle. The timestamps *t*_1_, *t*_2_, ..., *t*_n_ represent the absolute time for R peak used to calculate RR interval as shown in Equation 1. These *RR*_R_ values are used for the prediction of *RR*_P_ for the next time point *t*_10_. Previous *RR*_R_ intervals composing a full respiration cycles are also shown at the subsequent time points *t*_10_ to *t*_13_ (gray lines). A zoomed section is showing the predicted *RR*_P_ interval for the time point *t*_10_, given the present time point *t*_9_ using **(b)** the extrapolation method and **(c)** the interpolation method.

This method accounts for respiration-induced variability in *RR*_R_ intervals when predicting the future cardiac cycle. The predicted next RR interval $RR_{\text{P},\text{n}+1}^{3}$ (Equation 5) is obtained by adding a respiration-dependent correction term to the current *RR*_R_. The correction term is extrapolated from the *RR*_R_ values in the circular storage buffer, representing a single, full, and a preceding respiration cycle (Figure 2a) with *N* values of *RR*_R_. This term equals the slope of *RR*_R_ at the beginning of the stored respiration cycle multiplied by the average *RR*_R_ over the respiration cycle (Equation 4) to produce an extrapolated estimate of how the RR interval is likely to evolve (Figure 2b). Specifically, the slope is given as the difference between two consecutive RR intervals *RR*_R, n − N+1_−*RR*_R, n − N_ divided by the time difference of their corresponding R-peaks (*t*_R, n − N+1_−*t*_R, n − N_) (Equation 5, Figure 2b).


$$\begin{array}{l} \bar{t}=\frac{\left(t_{\text{R},\text{N}+1}-t_{\text{R},1}\right)}{N} \end{array}$$ (4)

$$\begin{array}{l} RR_{\text{P},\text{n}+1}^{3}=RR_{\text{R},\text{n}}+\left[\left(\frac{RR_{\text{R},\text{n}-\text{N}+1}-RR_{\text{R},\text{n}-\text{N}}}{t_{\text{R},\text{n}-\text{N}+1}-t_{\text{R},\text{n}-\text{N}}}\right)\cdot \bar{t}\right] \end{array}$$ (5)

The size of *N* is adaptively determined by continuously monitoring the RSA and detecting its consecutive minima corresponding to a single respiratory cycle. The number of discrete cardiac beats (i.e., the number of *RR*_R_) occurring between these two minima defines the instantaneous size of *N*, which is then continuously updated and stored in a circular buffer with each cardiac cycle. The storage buffer is continuously updated with each cardiac cycle. A schematic illustration of the extrapolation method is shown in Figure 2b, where the *RR*_P_ is estimated as a function of instantaneous slope and the average RR as $\bar{t}$ interval for one preceding and full respiration cycle, containing an illustrative with *N* = 8 RR intervals enclosed.

#### 2.2.4 Interpolation

This method also considers *RR*_R_ variation due to respiration and predicts accordingly. The predicted $RR_{\text{P},\text{n}+1}^{4}$ is computed by adding a respiration-dependent correction term to the current *RR*_R, n_. The correction term is interpolated from *RR*_R_ values in the circular storage buffer. Specifically, the correction term equals the linear interpolation value *RR*_interp_ of the stored respiration cycle (Equation 6) at the time point of the average *RR*_R_ (Equation 4), starting from the beginning of the cycle (Figure 2c), less the starting RR, *RR*_R, n − N_ of the respiration cycle (Equation 7).


$$\begin{array}{l} RR_{\text{interp}}=RR_{\text{R}}\left(\bar{t}\right) \end{array}$$ (6)

$$\begin{array}{l} RR_{\text{P},\text{n}+1}^{4}=RR_{\text{R},\text{n}}+\left[\;RR_{\text{interp}}-RR_{\text{R},\text{n}-\text{N}}\right] \end{array}$$ (7)

The interpolation method is depicted in Figure 2c, for one respiration cycle, (depicted in this specific example with *N* = 8 enclosed intervals).

The predicted *RR*_P_ is compared with the real *RR*_R_ and the performance of different methods is analyzed, upon computation of the *RR*_P_ by using the four different methods,. The error Δ*RR* between *RR*_R_ and *RR*_p_ is calculated using Equation 8 and explored further for accuracy in prediction.


$$\begin{array}{l} \Delta RR=RR_{\text{P}}-RR_{\text{R}} \end{array}$$ (8)

### 2.3 Statistical analysis

Statistical analysis was performed to evaluate the reliability and accuracy of the prediction methods for offline and real-time settings. Bland-Altman plots showed the dependence of Δ*RR* as a function of the mean of *RR*_P_ and *RR*_R_. The Anderson-Darling test was utilized to assess the normality of Δ*RR*. Due to the non-normal distribution of the data, non-parametric statistical tests were employed to assess the statistical difference between the methods. The accuracy of the prediction methods was quantified using absolute median error (MdAE), its interquartile range (IQR) at the 25^th^ and 75^th^ percentiles and the absolute median relative error MdRE of Δ*RR*_R_ (Equation 9) as measures of dispersion.

$$\begin{array}{l} \Delta RR_{\text{R}}=\frac{\left|RR_{\text{P}}-RR_{\text{R}}\right|}{RR_{\text{R}}}\;.\;100\% \end{array}$$ (9)

The relative error MdRE adjusts the prediction error Δ*RR* for the varying size of the instantaneous RR, specifically, its decrease during inspiration and increase during expiration.

To evaluate relative performance differences among the prediction methods, pairwise comparison was conducted using the Wilcoxon signed-rank test with a significance error probability of 0.05. A Bonferroni correction was applied for multiple testing to adjust the significance error level and avoid false positives.

## 3 Results

This section presents the results of the analyses conducted to verify the four different prediction methods for the future cardiac cycle estimation. Discrete offline analysis of the prediction methods is reported first, followed by analysis in a real-time closed-loop assembly of cardiac-gated aVNS.

### 3.1 Offline setting

**Figure 3:**
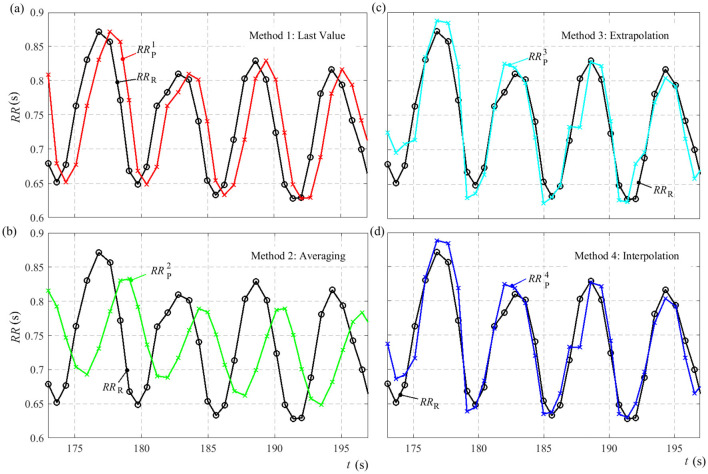
The time course of the predicted *RR*_P_ interval values in comparison with the true *RR*_R_ values for deep breathing in the offline setting, for the method of **(a)** last value, **(b)** averaging, **(c)** extrapolation, and **(d)** interpolation.

**Figure 4:**
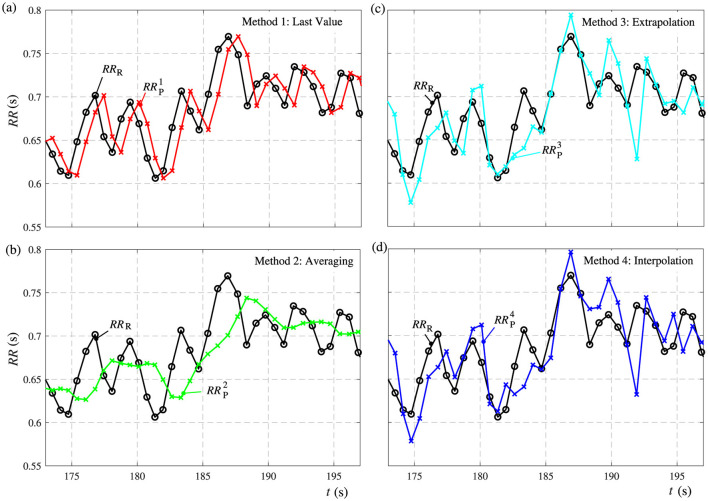
The time course of the predicted *RR*_P_ interval values in comparison with the true *RR*_R_ values for normal breathing in the offline setting (data from an in-house subject) for the method of **(a)** last value, **(b)** averaging, **(c)** extrapolation, and **(d)** interpolation.

**Figure 5:**
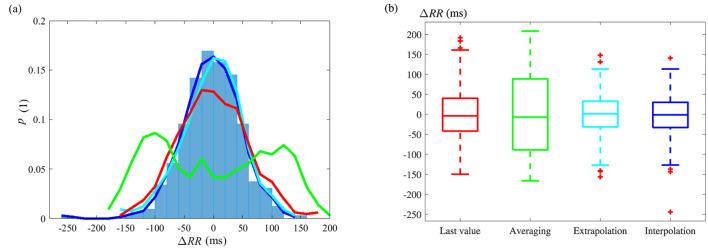
Prediction error Δ*RR* (Equation 8) between the predicted *RR*_P_ and real *RR*_R_ in case of paced deep breathing for all methods and offline settings. **(a)** Histogram of Δ*RR* with the included bars shown for the interpolation method. **(b)** Box plot of Δ*RR*.

**Figure 6:**
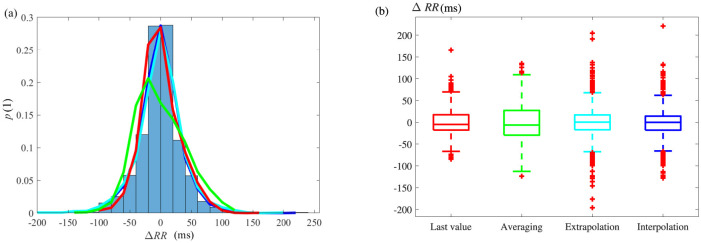
Prediction error Δ*RR* (Equation 8) between the predicted *RR*_P_ and real *RR*_R_ in case normal breathing (aggregated data from all three in-house subjects) for all methods and offline settings. **(a)** Histogram of Δ*RR* with the included bars shown for the interpolation method. **(b)** Box plot of Δ*RR*.

**Figure 7:**
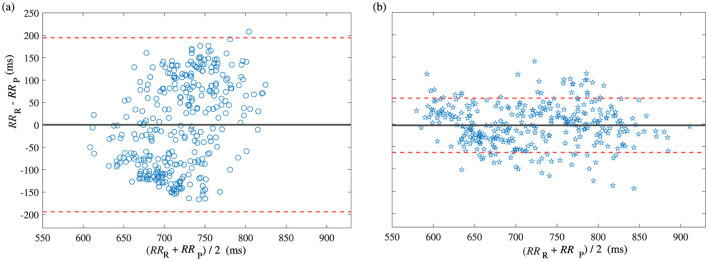
Bland-Altman plots comparing prediction errors during paced deep breathing between *RR*_P_ and *RR*_R_ for **(a)** Averaging, **(b)** Interpolation methods. The interpolation method yields narrower 95% limits of agreement than the averaging method.

**Figure 8:**
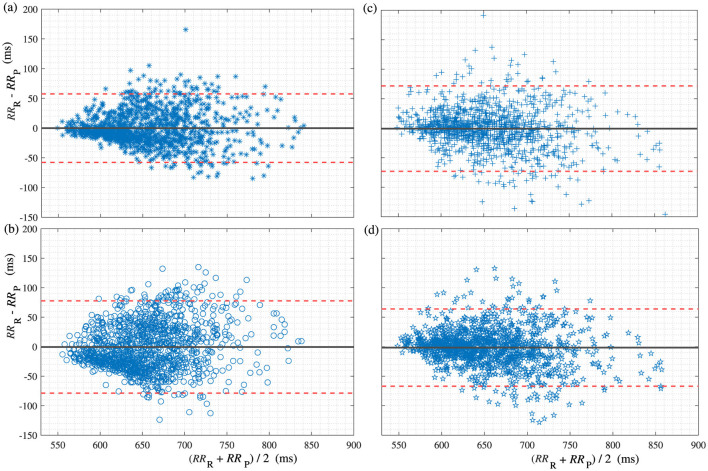
Bland-Altman plots for normal breathing (aggregated data from all three in-house subjects) between *RR*_P_ and *RR*_R_ for **(a)** Last value, **(b)** Averaging, **(c)** Extrapolation and **(d)** Interpolation methods.

Figures 3, 4 show the time courses of the true *RR*_R_ along with the predicted next *RR*_P_ derived from the previous true *RR*_R_ during offline analysis for paced deep breathing and normal breathing, respectively. This enables a visual qualitative comparison of prediction accuracy across the different prediction methods and breathing conditions. The quantitative variability Δ*RR* of the predicted *RR*_P_ is shown using histograms and box plots, for all four methods, in Figure 5 (paced deep breathing) and Figure 6 (normal breathing; aggregated from three independent 5-min offline recordings), respectively. Figures 7, 8 evaluate the agreement between the true *RR*_R_ and the predicted *RR*_P_ using Bland-Altman plots for paced deep breathing and normal breathing, respectively.

Most error distributions of the prediction methods, both for normal and deep breathing, violated the assumption of normality (*p* < 0.05), thus non- parametric statistical tests were utilized. Table 1 presents the MdAE, the MdRE, the IQR, and the pairwise statistical differences of the various prediction methods. During deep breathing, the extrapolation and interpolation methods were more reliable, with MdAE values of 32.09 ms and 31.71 ms, and MdRE values 4.59% and 4.55%, respectively. In contrast, the last value averaging methods resulted in substantially higher errors (MdAE values- 40.85 ms and 88.75 ms, and MdRE MdRE values 5.87% and 12.44% respectively). A priori, Wilcoxon signed-rank tests with Bonferroni correction (*p* < 0.0083) - the number of unique pairs amounts to 6, yielding the significance error of 0.05/6 = 0.0083 - confirms that both proposed respiration-sensitive methods were significantly different from the respiration-insensitive methods. There is no statistical difference between the two respiration-sensitive methods. Conversely, during normal breathing, extrapolation and interpolation yielded MdAE of 17.13 ms, and 16.46 ms, and MdRE 2.64% and 2.56% closely followed by the last-value method with MdAE 17.5 ms and MdRE 2.67%. As indicated by the significant markers, all three of these methods achieved significantly lower prediction errors compared to the standard averaging method (28.5 ms) (Table 1).

**Table 1:** Absolute median error for the prediction of Δ*RR* (Equation 8), with the interquartile range of (25–75)%, and absolute median relative error MdRE (Equation 9) for the offline analysis during deep and normal breathing conditions (in-house cohort) and healthy individual data and tachycardia data (open-source cohort-PhysioNet).

Breathing	Methods	Last value ^†^	Averaging^*^	Extrapolation^‡^	Interpolation^§^
Deep (in-house)	Absolute median error (ms)	40.85^*‡§^	88.75^†‡§^	32.09^*†^	31.71^*†^
	Absolute median relative error (%)	5.87	12.44	4.59	4.55
	Interquartile range (ms)	(19.7–68.4)	(58.7–124.15)	(16.07–56.61)	(15.55–54.06)
Normal (in-house)	Absolute median error (ms)	17.5^*^	28.47^†‡§^	17.13^*§^	16.48^*‡^
	Absolute median relative error (%)	2.67	4.44	2.64	2.56
	Interquartile range (ms)	(8.13–31.4)	(15.3–43.7)	(7.08–36.44)	(6.96–34.18)
Normal (open-source)	Absolute median error (ms)	32.8^*§^	34.35^†‡§^	26.8^*§^	25.5^*†‡^
	Absolute median relative error (%)	3.77	3.84	3	2.90
	Interquartile range (ms)	(16–50.4)	(15.9–58.5)	(11.7–53.4)	(11–49.3) (11–49.3)
Tachycardia (open-source)	Absolute Median error (ms)	10^*‡§^	20^†‡§^	31.4^*†§^	28.7^*†‡^
	Absolute median relative error (%)	1.49	2.8	4.4	3.5
	Interquartile range (ms)	(10–40)	(7.5–52.5)	(10–136.7)	(10–80.1)

^†^ Last value vs. other methods with *p* < 0.0083 (= 0.05/6).

^*^ Averaging vs. other methods with *p* < 0.0083.

^‡^ Extrpolation vs. other methods with *p* < 0.0083.

^§^ Interpolation vs. other methods with *p* < 0.0083.

To validate the robustness of the predictive models across a broader and independent population, the offline analysis on open-source healthy cohort resulted in a similar performance trend as the in-house cohort (normal breathing). As detailed in Table 1, the MdAE of extrapolation and interpolation 26.8 ms and 25.5 ms, MdRE 3% and 2.9%, respectively is lower compared to the last value and averaging methods with MdAE 32.8 ms and 34.35 ms, and MdRE 3.77% and 3.8%. The Wilcoxon signed-rank tests revealed the last value method is significantly different from the averaging and interpolation method. Conversely, analysis of the open source tachycardia data revealed that the simple last value method yielded lowest error with MdAE 10 ms, MdRE 1.49% compared to extrapolation method with the largest MdAE 31.4 ms and MdRE 4.4%. Here Wilcoxon signed-rank tests revealed that every prediction method is significantly different from the other methods.

### 3.2 Real-time setting

**Figure 9:**
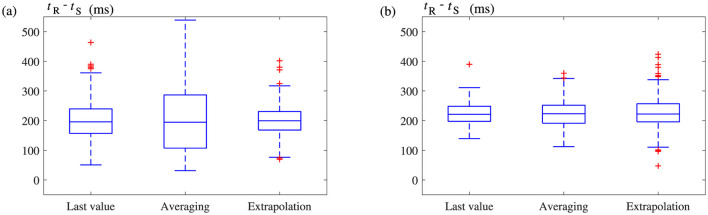
Box plots of *t*_R_−*t*_S_ illustrating the performance of the prediction methods in real-time that predict the next R-peaks and provide stimulation 200 ms before the predicted R-peak under **(a)** deep breathing and **(b)** normal breathing conditions.

Figure 1 illustrates segments of non-cardiac-gated (from Figure 1b) and cardiac-gated aVNS (from Figures 1d, f) recorded in real-time. The non-gated segment serves as a schematic demonstration for asynchronous aVNS stimulus delivery. For the cardiac-gated aVNS, aVNS is delivered relative to the predicted R-peak, i.e., either 200 ms before the R-peak during diastole (*t*_S_−*t*_R_ in Figures 1c, d) or 200 ms after the R-peak during systole ((*t*_S_−*t*_R_) < 0 in Figures 1e, f). The performance of three of the four methods, i.e., the last value, averaging and extrapolation, is illustrated in Figure 9 using box plots, which describe how well the next R-peaks are predicted in real-time during paced deep breathing and normal breathing for the aVNS to be delivered 200 ms before the R-peak.

Table 2 summarizes MdAE values and IQR for aVNS applied 200 ms before the predicted R-peak for paced deep breathing and normal breathing. During deep breathing, the extrapolation method yielded the highest predictive accuracy with an MdAE of 31.4 ms and MdRE 5.1%. In contrast, the last value and averaging methods resulted in higher errors of MdAE 41.8 ms and 88.0 ms, and MdRE 6.3% and 13.06% respectively. Significance levels for the real-time evaluations are also provided, utilizing the same pairwise Wilcoxon framework with a Bonferroni-adjusted threshold for the three methods (*p* < 0.0167 = 0.05/3). During deep breathing, all three algorithms demonstrated statistically significant differences from one another. Conversely, during normal breathing, the extrapolation method yielded an MdAE of 29.2 ms, and MdRE 4.6% followed closely by the last-value and averaging methods at MdAE 31.6 ms and 33.3 ms, and MdRE 4.3% and 4.9% respectively. The extrapolation method's performance converged with the averaging averaging method, yielding no statistically significant difference between the two (*p* = 0.49). During normal breathing, only the last-value method maintained a statistically distinct error distribution.

**Table 2:** Median absolute error for the prediction of the next R-peak ((*t*_R_−*t*_S_)−200 ms), with interquartile range of (25–75)% and absolute median relative error MdRE for real-time analysis; i.e., for aVNS stimulation 200 ms before the predicted R-peak (Real-time).

Breathing	Methods	Last value^†^	Averaging^*^	Extrapolation^‡^
Deep	Absolute median error (ms)	41.8^*‡^	88^†‡^	31.4^†*^
	Absolute median relative error (%)	6.3	13.06	5.1
	Interquartile range (ms)	(20.23–74.05)	(56.63–125.13)	(15.18–55.90)
Normal	Absolute median error (ms)	31.6^*‡^	33.3^†^	29.2^†^
	Absolute median relative error (%)	4.3	4.9	4.6
	Interquartile range (ms)	(13.3–49.37)	(14.62–55.12)	(16.3–61.4)

Deep and normal breathing conditions are considered.

^†^ Last value vs. other methods with *p* < 0.0167 (= 0.05/3).

^*^ Averaging vs. other methods with *p* < 0.0167.

^‡^ Extrapolation vs. other methods with *p* < 0.0167.

## 4 Discussion

It was found in our group that applying aVNS at different time points of the cardiac cycle can significantly affect the modulation of the resulting autonomic nervous system response (Tischer et al., 2024). Given the need to predict the duration of the next cardiac cycle for an optimal and flexible shift of aVNS stimulation within the cardiac cycle, predicted *RR*_P_ derived from the previous actual *RR*_R_ using last value, averaging, extrapolation, and interpolation methods has been proposed and assessed both in-house cohort and an independent open-source cohort.

### 4.1 Offline setting

The efficiency of the proposed methods, using the in-house cohort, for paced deep breathing (Figure 3) and normal breathing (Figure 4), demonstrates to what precision the *RR*_P_ follows the *RR*_R_. The RR intervals of ECG periodically fluctuate with the respiration cycle in line with the RSA, where the RR intervals narrow with inspiration and widen with expiration (Yasuma and ichiro Hayano, 2004; Kaniusas, 2012). Deep breathing, maximizes the amplitude of this RSA, creating the widest dynamic range of RR intervals and the sharpest beat-to-beat temporal variations. Successfully maintaining high predictive accuracy during the high physiological shifts of deep breathing would show the stability of our prediction methods. In particular, deep breathing, when combined with VNS, could lead to stronger activation of the parasympathetic networks, thereby maximizing neuromodulatory benefits of VNS (Garcia et al., 2017).

Thus, during the assessment of the prediction methods, we observe that, unlike the last value method, that takes the heart rate as an isolated time-series, and the averaging-based method, which diminishes (Figure 4b) or even fails (Figure 3b) to preserve the respiratory modulation of the RR intervals, the extrapolation and interpolation methods inherently take the heart and lungs as a coupled network, and use respiration-based correction terms (Equations 5, 7), for prediction. The state of one system in the network, the respiration, is used to predict the state of the other system, the cardiac activity.

Figures 5, 6 depict the range of deviation Δ*RR* of the predicted *RR*_P_ from the true *RR*_R_ during paced deep breathing and normal breathing, respectively. It suggests better cardiac cycle prediction through extrapolation and interpolation, accounting for dominant and cyclic changes in RR interval caused by respiration. The Bland-Altman analysis in Figure 7 shows superior performance of the interpolation method, with narrower 95% limits of agreement compared to the averaging method during paced deep breathing, indicating high predictive precision during high RSA.

In particular, compared to paced deep breathing (Figure 3), where the RR interval modulation due to respiration is more pronounced, normal breathing exhibits more randomness and less pronounced periodicity (Figure 4), increasing the difficulty in prediction. The statistical relationships shown in Table 1, highlight the advantage of respiration-sensitive prediction methods during dynamic physiological states. Respiration-insensitive methods (last value and averaging) inherently lag behind the signal during heightened RSA during deep breathing. The Wilcoxon analyses confirm that by calculating the immediate trajectory, the proposed extrapolation and interpolation methods successfully bypass this lag. The IQRs of these respiration-sensitive methods also indicate a significantly tighter clustering of prediction errors; e.g., interpolation gives an IQR of 15.55–54.06 ms, while averaging gives an IQR of 58.7–124.15 ms. This minimized dispersion is critical for cardiac-gated aVNS by restricting the maximum expected error margin. These prediction methods directly reduce the risk that stimulation triggers will drift outside the targeted therapeutic window during deep exhalations.

In contrast, during spontaneous normal breathing with its greater randomness and low variance state, the last value method performs statistically similarly to the more complex respiratory-sensitive methods. The averaging method, which filters out the beat-to-beat variations due to respiration and thus worsens the prediction of the next inter-beat interval, yields higher prediction errors. Furthermore, the IQR boundaries across the last value, extrapolation, and interpolation methods are highly comparable, with nearly identical error clustering. This suggests that when ambient RSA is low, the cardiac signal lacks the variance, the simple last value method is mathematically sufficient to prevent prediction overcompensation, yielding an equally stable and tightly bounded error distribution (Table 1). Bland-Altman plots during normal breathing (Figure 8) also show that the amplitude of RSA is attenuated, and that the limits of agreement of the averaging method are larger than those of the last value and interpolation.

The inclusion of the open-source cohort, successfully confirmed the generalizability of our primary findings. During normal resting conditions, the healthy cohort closely mirrored the in-house experimental data, with the proposed extrapolation and interpolation models maintaining slightly superior accuracy (MdRE: 3.0% and 2.90%, respectively) over the last value and averaging methods (MdRE 3.77% and 3.84% respectively). During abnormal heart rate from the open source tachycardia data, the simple last-value method achieved highly constrained accuracy (MdAE: 10 ms; MdRE: 1.49%), while the proposed extrapolation method heavily overcompensated, resulting in a severely widened IQR range (10–136.7 ms) and the highest relative error (MdRE: 4.4%). Tachycardic states are characterized by sympathetic dominance, which effectively blunts RSA, resulting in a rapid, rigid, low-variance, rather respiration-independent, and random cardiac rhythm. This makes the last value method the best option for prediction during tachycardia and the respiration-dependent methods rather inapplicable.

### 4.2 Real-time setting

Out of the four proposed methods, only three, the last value, averaging and extrapolation methods were implemented because of the hardware limitations. In particular, the three implemented methods are computationally effective and can be easily implemented in real-time using typical microcontroller-based hardware frameworks. In our proprietary hardware, execution had an latency of less than 100 ms (Dabiri et al., 2022). The interpolation method would require advanced computing capacities such as serial-to-parallel processing (e.g., FPGA or a multi-core microcontroller).

For deep breathing, Figure 9a portrays the smallest distribution width of the time shift *t*_R_−*t*_S_ between the aVNS stimulus and the R-peak for the extrapolation method, which clearly outperforms the averaging method. The superior performance of extrapolation, during deep breathing, is also confirmed by the statistical analysis in Table 2 that showed the extrapolation method not only achieved statistical separation from the respiratory-insensitive methods but also maintained a strictly constrained upper quartile boundary (15.18-55.90 ms for extrapolation in comparison with 56.63–125.13 ms for averaging), preventing massive predictive overshoots.

During normal breathing, presenting a low variance and highly random signal, all the methods exhibit comparable performance (Figure 9b). In Table 2, the last value method, only being statistically different from the other methods, proved highly effective for achieving mathematical distinction and the tightest overall IQR during normal breathing. The simple last value method also has the obvious advantage of a fast, straightforward implementation in real-time applications.

As depicted in Figure 9, the observed deviation of ±50 ms is roughly 5-10% of the entire cardiac cycle. Since aVNS targets rather systole or diastole period, which compose a significant fraction of 40-60% of the cardiac cycle rather than the exact milliseconds, this error deviation may not interfere with a correct placing of aVNS stimulus within the intended physiological window of systole or diastole.

This study proposes prediction methods, shows their feasibility, and pre-evaluates these methods in offline and real-time settings. Even though many sophisticated algorithms exist, like autoregressive models such as such as optimized ARIMA models (Shu, 2024) and Kalman-filter approaches (Hirao et al., 2026), for (Huang et al., 2020), with mean absolute error of 31 ms, to advanced learning (Staffini et al., 2022; Zacarias et al., 2024), that could predict heart rate variability dynamics, these are these are computationally expensive, their application is predominantly optimized for offline forecasting, and less suited for low power embedded aVNS stimulator. Therefore, prioritizing computational efficiency, our respiration-dependent RSA-based prediction methods achieve necessary hardware efficiency and excel in tracking cyclic cardiorespiratory modulation. Limitations, such as sudden autonomic shifts and extrasystoles, can lead to enlarged prediction errors. Nevertheless, the prediction methods calibrate upon detection of the next cardiac cycles of the next respiration cycle. Certain other limitations apply, such as the need to collect data from a larger number of people, especially, in real-time settings, and for varying physiological conditions (e.g., rapid breathing), especially potential aVNS patient cohorts with active aVNS (potentially affecting the predictability) should be included in future studies. Ultimately, these real-time boundary findings strongly support a hybrid, adaptive system architecture that may rely on respiration-insensitive methods during resting conditions and selectively engages respiration-sensitive extrapolation during heightened RSA. In addition, the proposed prediction methods are prone to further optimization.

This work is deeply rooted to the dynamic interactions between diverse physiological systems (Abreu et al., 2023). Likewise, we investigate how the instantaneous cardiac activity is affected by and can be predicted from respiration, both activities being modulated by the autonomic nervous system and forming a dynamic and highly personalized network (Cairo et al., 2023). The respiration-sensitive prediction of the future interbeat interval allows us for a precise time-gated aVNS and thus for a closed-loop real-time personalized aVNS. By restoring autonomic balance in real-time, this approach offers a robust pathway to prevent disease progression in both healthy individuals and pathological conditions such as chronic pain or epilepsy.

## 5 Conclusion

Personalized aVNS gains relevance with the electrical stimulus applied at a specific time point within the cardiac cycle, for which prediction of the next cardiac cycle is necessary. The present study proposes and evaluates the feasibility of the four different prediction methods, respiration-dependent and respiration-independent methods, shows their feasibility and offers their preliminary evaluation. An offline analysis reveals that the interpolation method provides best accuracy for R-peak prediction compared to other methods - last value, averaging and extrapolation, both in normal and paced breathing scenarios, thereby accounting explicitly for the cyclic influence of respiration on the RR intervals and making accurate predictions accordingly. During the initial method validation stage, performed in real-time on hardware, the extrapolation method yielded the best accuracy during deep breathing. During normal breathing, however, the accuracy of all the methods were comparable, suggesting the use of the simple last value method, which is easy to implement and fast in operation. These prediction methods would further be used to provide personalized aVNS, advocating consistent therapy, lower energy demand of stimulation and may mitigate adverse effects.

Future work will involve administering personalized aVNS to human subjects, following evaluation of their physiological parameters.

## Data Availability

The raw data supporting the conclusions of this article will be made available by the authors, without undue reservation.
